# Multilocus species trees and species delimitation in a temporal context: application to the water shrews of the genus *Neomys*

**DOI:** 10.1186/s12862-015-0485-z

**Published:** 2015-09-29

**Authors:** Javier Igea, Pere Aymerich, Anna A. Bannikova, Joaquim Gosálbez, Jose Castresana

**Affiliations:** Institut de Biologia Evolutiva (CSIC-Universitat Pompeu Fabra), Passeig Marítim de la Barceloneta 37, 08003 Barcelona, Spain; Department of Plant Sciences, University of Cambridge, Cambridge, CB2 3EA UK; Departament de Biologia Animal, Universitat de Barcelona, Avinguda Diagonal 643, 08028 Barcelona, Spain; Department of Vertebrate Zoology, Lomonosov Moscow State University, Leninski Gory 1, Moscow, 119991 Russia

**Keywords:** Gene flow, Introns, Mutation rates, Relaxed clock, Speciation

## Abstract

**Background:**

Multilocus data are becoming increasingly important in determining the phylogeny of closely related species and delimiting species. In species complexes where unequivocal fossil calibrations are not available, rigorous dating of the coalescence-based species trees requires accurate mutation rates of the loci under study but, generally, these rates are unknown. Here, we obtained lineage-specific mutation rates of these loci from a higher-level phylogeny with a reliable fossil record and investigated how different choices of mutation rates and species tree models affected the split time estimates. We implemented this strategy with a genus of water shrews, *Neomys*, whose taxonomy has been contentious over the last century.

**Results:**

We sequenced 13 introns and cytochrome *b* from specimens of the three species currently recognized in this genus including two subspecies of *N. anomalus* that were originally described as species. A Bayesian multilocus species delimitation method and estimation of gene flow supported that these subspecies are distinct evolutionary lineages that should be treated as distinct species: *N. anomalus* (*sensu stricto*), limited to part of the Iberian Peninsula, and *N. milleri*, with a larger Eurasian range. We then estimated mutation rates from a Bayesian relaxed clock analysis of the mammalian orthologues with several fossil calibrations. Next, using the estimated *Neomys*-specific rates for each locus in an isolation-with-migration model, the split time for these sister taxa was dated at 0.40 Myr ago (with a 95 % confidence interval of 0.26 – 0.86 Myr), likely coinciding with one of the major glaciations of the Middle Pleistocene. We also showed that the extrapolation of non-specific rates or the use of simpler models would lead to very different split time estimates.

**Conclusions:**

We showed that the estimation of rigorous lineage-specific mutation rates for each locus allows the inference of robust split times in a species tree framework. These times, in turn, afford a better understanding of the timeframe required to achieve isolation and, eventually, speciation in sister lineages. The application of species delimitation methods and an accurate dating strategy to the genus *Neomys* helped to clarify its controversial taxonomy.

**Electronic supplementary material:**

The online version of this article (doi:10.1186/s12862-015-0485-z) contains supplementary material, which is available to authorized users.

## Background

Species tree approaches, which take coalescence into account, together with the use of multiple genes, have led to highly supported phylogenetic relationships of closely related species [1]. In addition, the use of multiple loci in species delimitation has increased rapidly over the last few years and are currently being applied to a large number of taxonomic problems [2–6]. Species delimitation has been particularly problematic in the past due to the use of different diagnostic criteria to identify the existence of distinct species. Under a general lineage or unified species concept, the only criterion used is that lineages have to evolve separately from other lineages to be considered species [7]. Recently developed methods can help determine which lineages have been evolving in isolation and, eventually, would correspond to species. For example, the IMa2 program, which uses an isolation-with-migration model, can estimate the amount of gene flow or migration rate between two lineages [5]. This rate can then be compared with a threshold value for the migration rate below which populations are evolutionarily independent, as suggested by population genetic theory [8]. On the other hand, the formal species delimitation test implemented in the BPP program allows posterior probabilities to be determined for one-species and two-species models successively applied to sister lineages within a phylogeny [2, 9]. These methods to identify distinct evolutionary lineages rely on the use of multiple loci and do not employ any kind of time calibration for the species delimitation tests. Even if the split time of two lineages or populations is not used in species delimitation methodology, it would be important to place speciation events within an accurate temporal framework. Thus, different speciation scenarios could be evaluated and the factors that promoted speciation could be disentangled.

Establishing an appropriate molecular clock for estimating split times implies a number of decisions that can greatly affect calibration. Although molecular dating has been extensively studied in the past in the context of gene trees, some of the issues that arise in species trees of closely related species have been less frequently addressed [10]. One possibility for dating a species tree could be to use internal calibration points (e.g., fossil data that can be assigned to specific branches). However, many groups of organisms may not have reliable calibrations. When internal calibrations are not available, two frequently followed strategies may lead to unreliable estimates. First, extrapolating mutation rates from other organisms, perhaps the most common approach, may be questionable since mutation rates have been found to be highly variable among different genomic regions and between lineages [11, 12]. The error in split time estimations can be more severe if only the rate of one locus, for example a mitochondrial gene, is used. Second, the use of secondary calibrations obtained from higher-order phylogenies may also be problematic if these calibration points are very recent and estimated from a gene tree due to the effect of gene coalescence, that is, to the fact that the coalescence of genes occurs prior to the divergence of populations. This phenomenon is particularly noticeable at shallow splits, where the difference between gene divergence and population divergence may be a large proportion of the branch lengths estimated from a gene tree [13, 14]. Given the problems associated with these approaches, an alternative strategy to date species trees when internal calibrations are not available in shallow species trees could be to estimate lineage-specific mutation rates for each locus under study. Specifically, substitution rates can be estimated from a higher-order phylogeny, where it is generally easier to find reliable calibration points, using a Bayesian relaxed clock analysis [15, 16]. For neutral markers, substitution rates estimated this way can be used as proxy of mutation rates [17, 18]. Therefore, mutation rates would be estimated in a first step and, subsequently, the dated species trees would be reconstructed using these rates. Nevertheless, the additional difficulty of estimating specific mutation rates from a higher-order phylogeny means that in many species tree studies this strategy is not used and only the topology and relative timings of the cladogenetic events (but not absolute times) are estimated [19–21].

**Fig. 1:**
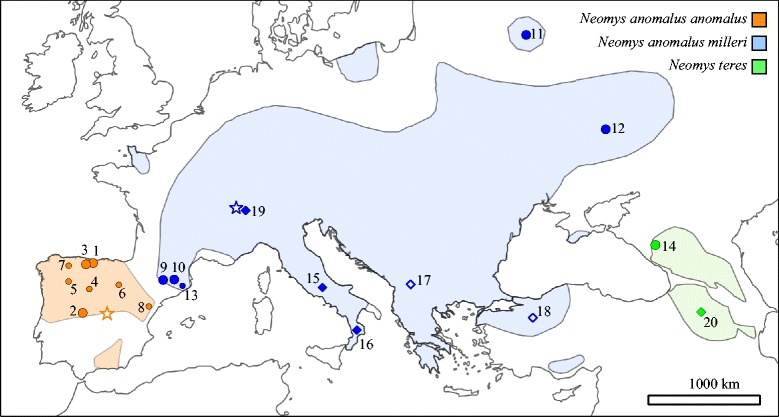
Map showing the distribution of *Neomys anomalus anomalus*, *N. a. milleri* and *N. teres*. The original distributions were downloaded from the IUCN Red List of Threatened Species website [72, 73] and they were modified to reflect the results of our genetic analyses. Dots indicate samples used for sequencing nuclear introns and cytochrome *b* (large dots) or only cytochrome *b* (small dots). The locations of additional cytochrome *b* sequences downloaded from databases are indicated with diamonds (with empty diamonds corresponding to samples being used in the cytochrome *b* tree but not in the species tree analyses). All localities correspond to a single specimen, except the localities of *N. teres*. Locality numbers are given in Tables S1 (for samples obtained for this work) and S2 (for database sequences) of Additional file 1. Type localities of *N. a. anomalus* (San Martín de la Vega, Madrid, Spain) and *N. a. milleri* (Chesières, Alpes Vaudoises, Switzerland) are indicated with a star symbol. The distribution of *N. fodiens* largely overlaps with that of the other species and it is not shown here; see Additional file 1: Tables S1 and S2 for further details

Multilocus species delimitation methods and dated species trees can be particularly useful for groups with contentious taxonomies. The water shrews of the genus *Neomys* are a case in point. This mammalian genus currently contains three recognized species [22]: *N. fodiens*, which has a Palaearctic distribution extending to Lake Baikal, with additional populations in eastern Russia and China; *N. teres*, which is present in the Caucasus and adjacent parts of Turkey and Iran; and *N. anomalus*, which has a patchy distribution over part of continental Europe and the Middle East. Two subspecies of *N. anomalus* (*N. a. anomalus* and *N. a. milleri*) were originally described in 1907 as separate species, by Cabrera [23] and Mottaz [24] respectively. Previously described morphological differences originally referred to the overall shape of the skull, rounded in *N. milleri* and more angular in *N. anomalus* [23, 24]. Further analyses revealed size differences in several measurements, particularly the length of the tail and hind foot, with *N. anomalus* being larger in both measurements [25–27]. However, these two taxa were cited as members of the same species in 1944 [28] and subsequent works followed the recognition of a single species [22, 29], without giving any clear argument for the regrouping. The known distribution of *N. a. anomalus* is centered in the Iberian Peninsula and *N. a. milleri* occupies the rest of the species’ Eurasian range (Fig. 1). All the species in this genus live in semi-aquatic habitats and usually feed underwater. Adaptations to aquatic life include stiff hairs on the tail and feet that aid in swimming and diving. These adaptations have been shown to be more prominent in *N. fodiens* [30] but, when these species live in sympatry, various instances of character displacement and convergence have been demonstrated [31, 32]. The phylogeny of this genus was studied using mitochondrial data, and *N. fodiens* was shown to be a sister group to the other species in the genus [33, 34].

Previous analyses with mitochondrial sequences of various European *Neomys anomalus* samples revealed a deep split between *N. a. anomalus* and *N. a. milleri* specimens [34]. We aimed to use multilocus data to test whether these two subspecies were sufficiently isolated to warrant species status, as originally described [23, 24]. For this purpose, we optimized 13 introns of nuclear encoded genes chosen from a set of highly variable sequence markers developed for mammals [35]. Using these sequences together with the mitochondrial cytochrome *b* gene*,* we applied different species tree reconstruction, gene flow estimation and species delimitation methodologies. The use of a high number of introns limited the number of samples to be used and therefore precluded any conclusion about the geographic variation of the genetic diversity. However, multiple loci allowed us, not only to apply modern multilocus species delimitation methods, but also to put the *Neomys* species tree in a temporal context. That is, we analyzed whether the main speciation events, and particularly those between sister taxa in the tree, took place during the Pleistocene or pre-Pleistocene epochs [36, 37] and whether more specific periods could be discriminated. To address these questions, we first estimated, from a mammalian tree, mutation rates specific for every intron in the *Neomys* lineage. We then calibrated the *Neomys* species tree with these rates. We also investigated how different choices of genes, species tree models (with and without gene flow) and mutation rates can affect the split time estimates.

## Methods

### Sample collection

Analyses were performed using 18 samples (Additional file 1: Table S1) from several specimens belonging to the three species currently recognized in the genus *Neomys*, with special emphasis on *N. anomalus*: *N. anomalus* (13 specimens, 7 of them with tissue samples and 6 with non-invasive samples), *N. teres* (2 specimens with tissue samples) and *N. fodiens* (3 specimens, 2 of them with tissue samples and 1 with non-invasive sample). The 11 tissue samples were used for sequencing multiple nuclear loci. These samples were obtained from specimens deposited in different biological collections as part of previous works, specimens found dead in the field in public areas, and specimens specifically captured for this work. All captures were performed with official permits obtained after the evaluation of a formal proposal by the corresponding nature conservation institutions (Additional file 1: Table S1). From the 7 non-invasive samples, which included faeces and a skull obtained from an owl pellet, only mitochondrial information could be obtained but they afforded a wider coverage of the likely contact zone between the two subspecies of *N. anomalus* in the Iberian Peninsula (Fig. 1). The collection localities are presented in Additional file 1: Table S1. Both Additional file 1: Table S1 and Fig. 1 reflect the assignment of *N. anomalus* specimens based on the subsequent genetic analysis. All samples were obtained according to relevant national and international guidelines for these species. All species are listed as “Least concern” by the International Union for Conservation of Nature (IUCN).

### DNA extractions

Total genomic DNA was extracted from tissue samples with DNeasy Blood and Tissue kit (QIAGEN) following manufacturer’s instructions, and using 75 μL of H_2_O for the final elution. For the skull obtained from an owl pellet, the lower mandible was first powdered using liquid nitrogen. The extraction was then performed as above, but the proteinase K incubation time was extended to 6 h to ensure maximum lysis of the tissue.

Fresh fecal samples attributed to *Neomys* were collected from different river localities using identification and conservation protocols previously established for another semiaquatic mammal [38]. The faecal DNA extractions were performed using the QIAmp DNA Stool Mini Kit (QIAGEN). The final elution volume was, in this case, 50 μL.

For the mandible and fecal samples, extractions were performed in a separate room with UV irradiation where no fresh tissue extraction had been performed, and dedicated equipment which was cleaned with a bleach-containing solution to prevent contamination. Extraction blanks were included so that any possible contamination could be detected at each step. Additionally, pre-PCR set up was carried out in a dedicated UV-hood in a controlled, sterile room.

### PCR amplification and sequencing of cytochrome *b*

Using representative cytochrome *b* sequences of the genus *Neomys* obtained from GenBank (Additional file 1: Table S2), we designed specific primers for the cytochrome *b* gene (Additional file 1: Table S3). The PCR conditions were as follows: an initial denaturation of 3 min at 95 °C, followed by 34 cycles of denaturation (30 s at 95 °C), annealing (30 s at 54 °C), and extension (60 s at 72 °C). The PCR reactions were performed in a final volume of 25 μL containing 2–4 μL genomic DNA, 0.75 units of Promega GoTaq DNA polymerase and 1 μM of each primer. For fresh tissue DNA extractions, the whole cytochrome *b* sequence (1140 bp) was obtained in a single PCR reaction using the flanking primers. For more degraded DNA samples, three overlapping PCR fragments were amplified. PCR products were purified using ExoSAP-It (Affymetrix) and sequenced using the PCR primers at Macrogen Inc (Seoul, South Korea). Sequences were subsequently inspected and assembled using Geneious Pro (Biomatters Ltd).

### Phylogenetic analyses of cytochrome *b*

We performed a phylogenetic analysis of the 18 complete cytochrome *b* sequences we obtained and of 14 additional *Neomys* sequences downloaded from GenBank (Additional file 1: Table S2). Two of them were shorter sequences (between 350 and 400 bp) of *N. a. milleri* from Macedonia and Turkey and were included to ensure a greater coverage (these two sequences were used in the cytochrome *b* tree reconstruction but not in the species tree analyses). Moreover, the cytochrome *b* sequences of four specimens of the *Chimarrogale* genus were used as outgroups since they are closely related to *Neomys* [39, 40] (Additional file 1: Table S2). Using these sequences, a multiple sequence alignment was built using MAFFT version 7.130 [41]. A maximum-likelihood tree was calculated with RAxML version 7.4.2 [42] using a general time reversible (GTR) substitution model and rate heterogeneity modeled with a Gamma distribution plus a proportion of invariable sites, as suggested by jModeltest version 2.1.5 using the Akaike information criterion [43]. Node support was assessed by performing 100 bootstrap replicates. In addition, a Bayesian inference phylogenetic tree was built using BEAST version 1.8 [44]. The GTR model suggested by jModeltest did not converge and therefore we used the second best model: Hasegawa-Kishino-Yano (HKY) [45] with a proportion of invariable sites. A strict molecular clock was chosen and a coalescent constant-size model was used as tree prior. See Additional file 1 for further details.

### PCR amplification and sequencing of nuclear markers

Thirteen nuclear introns were selected from a set of phylogenetic markers that had previously been developed for mammals [35] (Additional file 1: Table S4). Using flanking exon information provided by Igea et al. [35] as well as additional orthologous exon data downloaded from the ENSEMBL database [46], we designed primers in the most conserved regions (Additional file 1: Table S4). After sequencing, the haplotypic phases of the introns were determined using PHASE version 2.1.1 [47], with a threshold of 0.9. The remaining unresolved haplotypes were cloned into a pSTBlue vector-1 (Invitrogen) according to the manufacturer’s instructions and between three and five clones per PCR fragment were sequenced.

The 13 nuclear loci and their corresponding alleles were successfully sequenced in all the *Neomys* tissue samples except one, where the alleles of only 8 introns could be obtained. Most of the introns were also amplified in two additional soricid species (*Sorex coronatus* and *Crocidura russula*) to complete the mammalian data set for substitution rate estimation.

The *Neomys* alleles of each intron were aligned with MAFFT [41] with default options and all gap positions were removed for further analyses. The absence of recombination in the 13 nuclear loci was checked using the Φ test [48]. A maximum-likelihood tree was reconstructed with RaxML version 7.4.2 [42] from each intron alignment as described before. From each of these trees, a haplotype genealogy was generated using Haploviewer 1.0 [49], which groups identical sequences in circles of size proportional to the number of sequences, colors circles by group, and plots mutations in branches as estimated from the corresponding alignment.

### Group assignment with nuclear genomic trees

In order to help in the group assignment of the different *Neomys* specimens, and particular those of *N. anomalus*, which are the most problematic, we estimated the average genetic relationship between the specimens using two different phylogenetic methods. First, we reconstructed a distance tree from a matrix of the average genomic divergence between the *Neomys* individuals [50, 51]. Because nuclear genomes are diploid, a method is needed to summarize the divergence of the two separate alleles per individual. For this, we calculated pairwise distances between all specimens using the formula 8.2 in Freedman et al. [51]. Distances were corrected for multiple substitutions using the Jukes-Cantor formula. The resulting matrix of pairwise distances was used to reconstruct a distance tree using the Fitch program of the Phylip package [52], which has the advantage over the neighbor-joining method of not allowing negative branches. See Additional file 1 for further details.

In addition, we reconstructed a nuclear genomic tree by concatenating all introns. Since there are two alleles per intron (most of the times of different sequences), the order of concatenation of the two alleles may affect the results. We therefore performed several concatenations in which the order of each allele pair was randomly changed. From each concatenation set, a maximum-likelihood tree was reconstructed with RaxML version 7.4.2 [42] using a GTR plus invariable sites model, as described before.

### Number of groups and group assignment with Structurama

To further help in the placement of different specimens in different groups, we used Structurama version 2.0 [53, 54], which allows estimating the number of groups with highest probability and performs a population assignment test using frequencies of the nuclear data. In a first run, the number of populations was set as a random variable using either a Dirichlet process prior with different alpha values or with the alpha value allowed to be a random variable with a gamma probability distribution set as: (1, 1). In a second run, we fixed the population number with highest probability and determined the group assigned with highest probability for each specimen. The same assignment was determined when an admixture model was used (not shown). See Additional file 1 for further details.

### Estimation of nuclear DNA substitution rates: BEAST

In order to obtain an accurate estimate of the substitution rates of the 13 introns, we carried out a Bayesian relaxed clock analysis using the intron sequences from *N. fodiens* and the two additional soricid species sequenced by us (*S. coronatus* and *C. russula*), together with orthologous sequences from other mammalian orders downloaded from ENSEMBL [46]. Not all the introns were available for all of the selected species, resulting in 13 % missing data in the final dataset. The multiple sequence alignments were obtained using MAFFT version 7.130 with the recommended high accuracy options [41]. Gblocks version 0.91 with relaxed parameters [55] was then used to eliminate any poorly aligned regions, primarily consisting of some large insertions in certain sequences. Gblocks with relaxed parameters eliminates blocks of non-conserved positions and positions with gaps in more than 50 % of the sequences. Therefore, this cleaning procedure eliminated the large insertions while maintaining the core of the intron alignments. The 13 resultant cleaned intron alignments were included as independent partitions in the Bayesian relaxed clock analysis performed with the program BEAST version 1.8 [16, 44]. The best fitting nucleotide substitution model suggested by jModeltest [43] was a GTR and Gamma model for most introns and it was used for all partitions. An uncorrelated lognormal relaxed molecular clock was applied to each partition. Using the data provided in Benton et al. [15], several mammalian fossil dates were used as hard bound minimum and soft bound maximum constraints in eight key nodes in order to calibrate the phylogenetic tree. These nodes are the splits between several divergent lineages of mammals for which genome sequences are available (Additional file 1: Table S6) and the calibrations have been highly curated for Bayesian dating analysis [15]. The substitution rates of each intron averaged across all lineages were obtained from the log file using the program Tracer of the BEAST package. Specific *Neomys* rates for each intron were extracted from the posterior distribution of trees. In this large file, the rates of each intron are associated to each branch in the description of each tree and were extracted with a custom-made script. From the distributions of substitution rates, we obtained the mean and standard deviation values. The original alignments, with no Gblocks cleaning, rendered basically the same substitution rates (not shown). See Additional file 1 for further details.

### Estimation of mitochondrial DNA substitution rate: BEAST

Since the high substitution rate of the cytochrome *b* is likely to be saturated in a tree of mammalian orders [56], the estimation of this rate was performed with a set of soricid species. Complete cytochrome *b* sequences belonging to 57 soricid species were downloaded from GenBank and a Bayesian relaxed clock analysis was carried out using BEAST [16, 44]. A GTR substitution model and rate heterogeneity modeled with a Gamma distribution and a proportion of invariable sites was used, as suggested by jModeltest [43]. In order to try to overcome the effect of saturation, the analysis was partitioned by codon position. An uncorrelated lognormal relaxed molecular clock was applied. The tree was calibrated using a set of fossil constraints available for soricids (Additional file 1: Table S7), previously used in this group [39], setting lognormal prior distributions as before. The program TreeAnnotator from the BEAST package was used to obtain a maximum clade credibility tree, from which the substitution rate in the *Neomys* branch was extracted. See Additional file 1 for further details.

### Species tree estimation: *BEAST

The differentiated groups found in the genome tree and Structurama test were set as independent populations in *BEAST [57]. Thirteen nuclear introns and the cytochrome *b* sequences from 11 *Neomys* specimens were used in addition to cytochrome *b* from 19 additional specimens either sequenced by us or obtained from GenBank, to better cover the genetic variability within each population (Additional file 1: Tables S1 and S2). To estimate the topology of the groups, the program was first used with relative mutation rate priors (meaning that the scale of the species tree will be in mutations rather than years). We gave a normal prior distribution with a mean rate of 1 and a standard deviation of 0.5 to all the introns. In this analysis, the mean prior for the cytochrome *b* mutation rate was set 10 times higher than the rate for introns to account for the fact that approximately a 10 times faster rate has been found for mitochondrial sequences with respect to intron sequences [35], using a lognormal distribution with a standard deviation of 1. The long tail of high values of this prior lognormal distribution accounts for the possibility that the proportion of cytochrome *b* rate with respect to the intron rates was underestimated.

For the dating analysis, a new *BEAST run was performed with mutation rates. We considered substitution rates previously estimated from our intron and mitochondrial mammalian trees as mutation rates to be used in the species tree, under the assumption that the markers are neutral and selection did not significantly affect them [17, 18]. In this analysis, normal distributions with the means and standard deviations of the rates that had been previously calculated in the mammalian multilocus analysis were used as priors for the mutation rates of the 13 introns. For cytochrome *b*, a lognormal distribution was set as prior (with the mean taken from the cytochrome *b* analysis of Soricidae and a standard deviation of 1) to account for the possibility that the mutation rate previously calculated was saturated. See Additional file 1 for further details.

### Isolation-with-migration model: IMa2

The IMa2 software (version updated 27/8/2012) [58] was used to fit the sequence data to an isolation-with-migration model, using the same intron alignment data as above. This Bayesian program estimates parameters related to population size and divergence time but, unlike *BEAST, allows for the presence of gene flow among the studied taxa. However, the phylogenetic relationship between the populations has to be established *a priori*; hence, the resultant topology from *BEAST was used. Preliminary analyses revealed that the data did not contain enough information to estimate all the ancestral migration parameters. Thus, the model was constrained so that gene flow could only take place between the three closest present-day lineages, that is, *N. teres* and the two subspecies of *N. anomalus*. The program output was used to obtain the migration rate divided by the mutation rate per generation (m/μ) and the effective number of migrant genes per generation (2Nm) along with the effective number of migrant individuals per generation (Nm). We express the direction of migration forwards in time. A likelihood ratio test was performed to assess whether the migration rates differed from zero. Other parameters in IMa2 are given in mutations per locus and are proportional to the geometric mean of the mutation rates per locus. Therefore, we calculated mutation rates per locus by multiplying the previously estimated mutation rates by the length of the *Neomys* alignments after removing gap positions. The geometric mean of the mutation rates per locus of the 14 loci was used to convert split times and effective population sizes to demographic units. See Additional file 1 for further details.

### Bayesian species delimitation: BPP

To assess whether the four *Neomys* lineages corresponded to species, we used the Bayesian species delimitation program BPP version 2.2 [2]. The topology from the *BEAST analysis was used as a guide tree; starting from this tree, BPP collapses sister lineages when they best fit a one-species model. The introns and the cytochrome *b* sequences, or only the introns, were used as independent loci. Twenty different prior schemes and algorithms were employed (see Additional file 1) as these have been shown to affect the results in certain circumstances [9].

## Results

### Mitochondrial phylogeny of *Neomys*

**Fig. 2:**
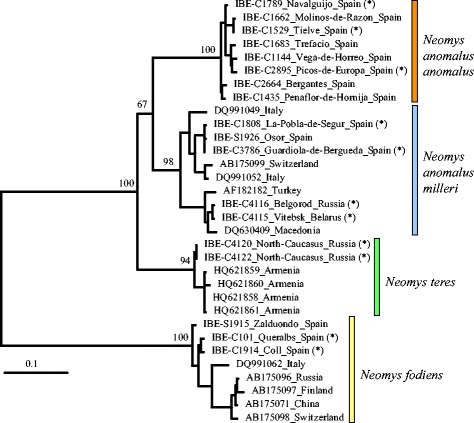
Maximum-likelihood phylogenetic tree of the cytochrome *b* sequences of the genus *Neomys* with bootstrap values shown for the nodes connecting all linages of each species or subspecies and their splits. Four species of *Chimarrogale* were used as outgroup (not shown). The scale bar is in substitutions/site. Names include specimen code and locality data. Names followed by a star symbol indicate samples that were used for intron sequencing

The maximum-likelihood mitochondrial phylogeny of *Neomys* (Fig. 2) showed that the *N. anomalus* sequences were divided into two divergent lineages (6.8 % average observed differences), which formed a weakly supported clade (67 % bootstrap). The same clade was found in a Bayesian tree (not shown), with a posterior probability of 0.92. Interestingly, the first of these lineages grouped together the northeastern Iberian samples with the other European samples, while the second one contained the remaining Iberian samples. Therefore, the two lineages largely correspond to the two described subspecies of *N. anomalus*: *N. a. milleri* and *N. a. anomalus*. The only difference was found in the northeastern Iberian samples, which historically have been grouped together with the other Iberian samples as *N. a. anomalus* [59], but here were found to cluster with the non-Iberian European samples. Thus, we preliminarily assigned the samples from northeastern Iberia to *N. a. milleri*. The mitochondrial sequences of *N. teres* and *N. fodiens* agreed with previous species determinations (Additional file 1: Table S1).

### Nuclear data

**Fig. 3:**
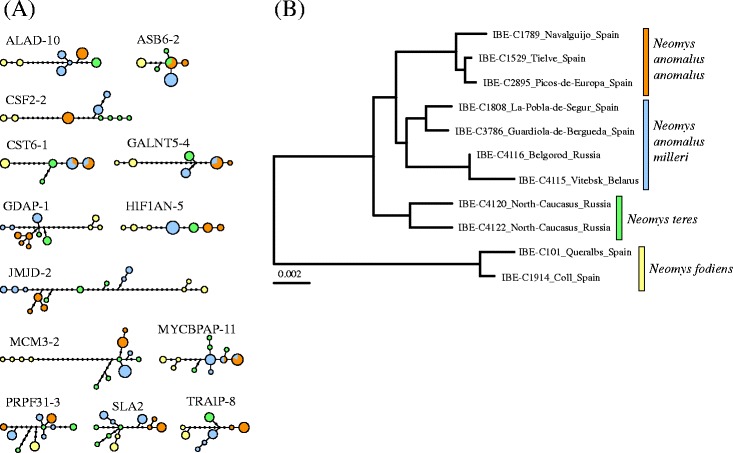
**a** Haplotype genealogies of the 13 introns amplified in the genus *Neomys*. The sizes of the circles are proportional to the number of haplotypes detected. **b** Distance tree based on an estimate of the average genomic divergence between the introns of *Neomys* specimens. The tree was rooted at the midpoint. The scale bar is in substitutions/site

Thirteen intron markers were sequenced from each of the 11 *Neomys* specimens with tissue samples available. The number of alleles determined for each lineage was 78 for *N. a. anomalus,* 94 for *N. a. milleri*, 52 for *N. teres* and 52 for *N. fodiens*, totaling 129,494 bp of nuclear sequence information for all specimens together. From the intron alignments, the percentage of gaps removed previous to the analyses varied between 0.2 and 7.3 % of the alignment positions, with an average of 2.1 %. All introns were variable between the different species and showed some degree of intraspecific variation, as shown in the maximum-likelihood trees (Additional file 1: Figure S1) and the haplotype genealogies reconstructed from these trees (Fig. 3a). The four lineages preliminary considered in the mitochondrial tree were shown to constitute separated groups in four of the introns (ALAD-10, CSF2-2, HIF1AN-5 and TRAIP-8) but not in the others (Additional file 1: Figure S1 and Fig. 3a), making necessary further analyses of the nuclear data.

### Species trees and species delimitation

In order to test if the four *Neomys* species and subspecies analyzed here correspond to differentiated lineages, we used two different methods: 1) the estimation of migration rates using IMa2 [58] and their comparison with rates known to cause population differentiation, and 2) the species delimitation test implemented in BPP [2]. These two methods need *a priori* estimation of the initial number of groups, the assignment of specimens to the different groups and a topology relating these groups. Importantly, none of these estimates require knowledge of mutation rates of the loci under study.

#### Estimation of the initial number of groups and assignment of specimens to the different groups: Nuclear genomic tree and Structurama

Although mitochondrial data showed a clear subdivision between the four lineages, a hypothetical mitochondrial introgression may have caused an erroneous species assignment for some specimens. This issue could be particularly relevant for specimens close to the contact zone of the two *N. anomalus* lineages (i.e., those from northeastern Iberia). In order to analyze these lineages with the nuclear data, we first reconstructed a distance tree based on an estimate of the average genomic divergence of the introns of the 11 *Neomys* specimens from which multilocus data could be obtained (Fig. 3b). Despite the potential for deeper coalescence in the nuclear genes, this tree supports the same group assignment as the mitochondrial data and again shows the northeastern Iberian individuals to be more closely related to those from the rest of Europe. Very similar trees were obtained from the concatenation of the introns and the reconstruction of a maximum-likelihood tree. In 10 concatenations where the order of the two alleles of each intron was randomly changed, the resulting trees were similar among each other and similar to the tree obtained by the distance method (Additional file 1: Figure S2), therefore also supporting the same group assignment.

In addition, we conducted a group assignment test with Structurama [54] applied to the intron allele frequencies. We found that the number of populations with the highest posterior probability was 4 for all prior sets (Table 1). After fixing this number of populations, a new analysis was performed to determine the group assigned with highest probability for each specimen. In this analysis, the assignment of the 11 *Neomys* specimens to the four different groups was the same as that deduced from the mitochondrial and genome distance trees. We therefore maintained the four groups and the corresponding assignments. After the genome tree and Structurama test, we could assign three specimens to *N. a. anomalus* and four to *N. a. milleri* (Additional file 1: Table S1). In addition, since this assignment based on nuclear data was totally congruent with the mitochondrial tree (Fig. 2), we assigned the rest of specimens using this mitochondrial tree. Thus, 5 additional specimens were assigned to *N. a. anomalus* and 1 to *N. a. milleri* (Additional file 1: Table S1).

**Table 1:** Posterior probabilities of the number of populations (K) found by Structurama using the intron allele frequencies of *Neomys* specimens for different values of the prior mean of the number of populations (α)

	Posterior probability			
K	α = 3	α = 4	α = 5	α ~ gamma
1	0.00	0.00	0.00	0.00
2	0.00	0.00	0.00	0.00
3	0.00	0.00	0.00	0.00
4	0.78	0.65	0.52	0.69
5	0.20	0.31	0.40	0.27
6	0.01	0.04	0.08	0.04
7	0.00	0.00	0.00	0.00

#### Species tree estimation: topology

Using a species tree approach, the topology for the four detected lineages was estimated with *BEAST [57] using 14 independent loci corresponding to the 13 introns plus the cytochrome *b* gene*.* In this first analysis, only the topology was of interest. The resultant topology (Additional file 1: Figure S3) was compatible with that obtained in the mitochondrial and genome distance trees. However, the node grouping the two *N. anomalus* subspecies was more strongly supported, with 0.99 of posterior probability. The same strongly supported topology was seen when only introns were used.

#### Isolation-with-migration model and estimation of migration rates

The application of the IMa2 isolation-with-migration model [58] using the 13 introns and cytochrome *b* allowed us to estimate the level of gene flow between the different extant lineages of *Neomys*. Only the migration rate parameter m/μ from *N. a. anomalus* to *N. a. milleri* (m/μ = 0.183; 95 % confidence interval: 0.053 – 0.533) differed significantly from zero. This migration rate corresponds to Nm = 0.072 effective migrants per generation, which is well below the threshold known to cause population differentiation (Nm = 0.5) [8].

#### Species delimitation test with BPP

We also applied the Bayesian species delimitation method implemented in BPP [2], using the 13 intron markers and cytochrome *b*. Under all prior schemes, the tree with four differentiated lineages received a posterior probability higher than 0.95 (Table 2), indicating that these lineages could correspond to four distinct species, including the two *N. anomalus* subspecies. The same results were obtained when only introns were used (not shown).

**Table 2:** Combinations of priors (mean θ, mean τ, and α of the Gamma distribution) and rjMCMC algorithm used in the BPP analysis and posterior probability of the four-species tree

	Posterior probability			
Values for mean θ and τ priors	α = 2; algorithm 1	α = 2 algorithm 2	α = 20; algorithm 1	α = 20; algorithm 2
θ = 0.002 and	1	1	1	1
τ = 0.008				
θ = 0.0002 and	1	0.95	1	1
τ = 0.008				
θ = 0.02 and	1	1	1	1
τ = 0.008				
θ = 0.002 and	1	1	1	1
τ = 0.0008				
θ = 0.002 and	1	1	1	1
τ = 0.08				

### Split time estimation

In order to date the species tree of *Neomys* species we first used BEAST [44] to estimate the substitution rates of each locus. For this purpose, we performed two different Bayesian relaxed clock analyses, one for the introns and another one for the cytochrome *b* gene. These rates were then used to convert split time parameters obtained by IMa2 into units of million years (Myr) [58]. They were also used as priors in a new analysis of *BEAST [57] to estimate the split times of the species tree, for comparison with those obtained by IMa2.

#### Estimation of nuclear and mitochondrial substitution rates in mammals and Neomys

**Fig. 4:**
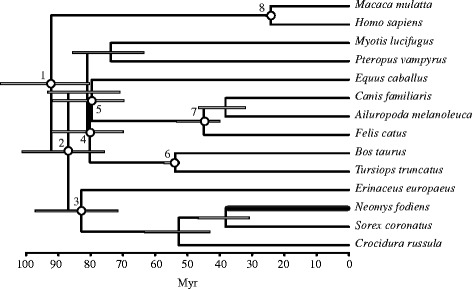
Bayesian relaxed clock tree reconstructed with mammalian intron sequences. Calibration nodes are shown with a white circle. The taxa corresponding to these nodes and the corresponding minimum and maximum constraints, in Myr, are: Boreoeutheria (1): 61.50 and 131.50; Laurasiatheria (2): 62.50 and 131.50; Eulipotyphla (3): 61.50 and 131.50; Ferungulata (4): 62.50 and 131.50; Zooamata (5): 62.50 and 131.50; Cetartiodactyla (6): 52.40 and 65.80; Carnivora (7): 39.68 and 65.80; and Catarrhini (8): 23.5 and 34.00. The *Neomys fodiens* branch from which mutation rates were estimated is shown with a thicker line

We calculated intronic substitution rates from the Bayesian relaxed clock analysis implemented in BEAST [44]. This analysis included the 13 introns used in this work together with several fossil calibrations (Fig. 4). The estimated mean of the *Neomys*-specific intronic substitution rate was 0.0060 substitutions/site/Myr but important differences in rates estimated for the different introns were found (Table 3). The average rate for all mammalian lineages was 0.0031 substitutions/site/Myr (Table 3). As a comparison, following the same approach, we obtained a substitution rate of 0.0015 substitutions/site/Myr for the human lineage. These results suggest an accelerated rate in the *Neomys* branch when compared to other mammals.

**Table 3:** Estimated mutation rates of introns and cytochrome *b* in the *Neomys* branch and in the overall tree. Intron rates were estimated from the mammalian tree and cytochrome *b* rates from the soricids tree. Standard deviations (S. D.) of the mutation rate in *Neomys* are also given. Units are in mutations/position/Myr

Marker	Mutation rate in *Neomys*	Mutation rate in the overall tree	S. D. in *Neomys*
ALAD-intron-10	0.0056	0.0030	0.0012
ASB6-intron-2	0.0125	0.0044	0.0038
CSF2-intron-2	0.0024	0.0021	0.0004
CST6-intron-1	0.0104	0.0042	0.0025
GALNT5-intron-4	0.0040	0.0022	0.0007
GDAP1-intron-1	0.0050	0.0024	0.0017
HIF1AN-intron-5	0.0026	0.0017	0.0005
JMJD-intron-2	0.0076	0.0046	0.0018
MCM3-intron-2	0.0056	0.0027	0.0011
MYCBPAP-intron-11	0.0045	0.0028	0.0009
PRPF31-intron-3	0.0059	0.0045	0.0013
SLA-intron-2	0.0094	0.0036	0.0043
TRAIP-intron-8	0.0031	0.0028	0.0007
Cytochrome *b*	0.0231	0.0217	

The substitution rate of the cytochrome *b* gene is too high (and prone to saturation) to be reliably estimated from a tree of mammalian orders [56]. We therefore used BEAST to reconstruct a dated tree of cytochrome *b* sequences from the Soricidae using a different set of fossil calibrations (Additional file 1: Figure S4). In this way the substitution rate obtained for cytochrome *b* in the *Neomys* branch was 0.0231 substitutions/site/Myr.

#### Dating the species tree

**Fig. 5:**
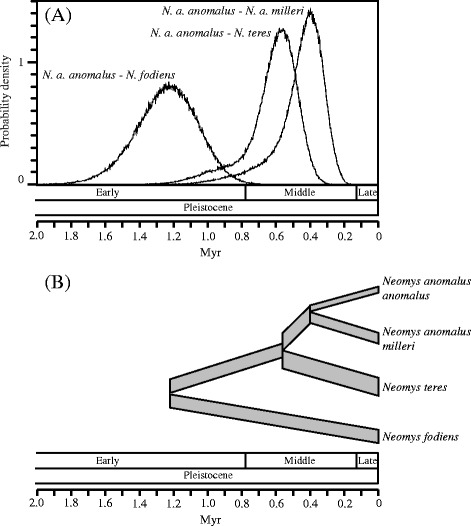
**a** Posterior probability density distributions for split time parameters corresponding to the isolation-with-migration model in IMa2. The X-axis was scaled with the geometric mean of the mutation rates to reflect time in Myr. The limits of the major ages of the Pleistocene are shown [69]. **b** Species tree of the *Neomys* lineages with split times and effective population sizes estimated by IMa2. The widths of the different population segments are proportional to the estimated effective population sizes. The X-axis is at the same scale than in (**a**)

Substitution rates previously calculated were then used as mutation rates to estimate split times with the isolation-with-migration model in IMa2. When the geometric mean of the mutation rates estimated for the 14 markers was used to scale the parameters, the divergence time between the two *N. anomalus* lineages was 0.40 Myr (95 % confidence interval: 0.26 – 0.86 Myr), while the split of these two lineages from *N. teres* was 0.56 Myr (0.40 – 1.04 Myr) and the divergence of *N. fodiens* with respect to the other lineages was 1.22 Myr (0.90 – 1.62 Myr) (Table 4 and Fig. 5a). Figure 5b represents the species tree with the corresponding split times and branch widths proportional to the effective population sizes.

**Table 4:** Split times (and 95 % confidence intervals) of *Neomys* calculated in a species tree approach with IMa2 and *BEAST using different combinations of genes and estimated mutation rates. For IMa2, the isolation-with-migration (IM) or the isolation (I) models were used. Results obtained with a direct gene tree approach of the mammalian introns using BEAST are also shown

	*N. a. anomalus* and *N. a. milleri* split (Myr)	*N. anomalus* and *N. teres* split (Myr)	*N. anomalus* and *N. fodiens* split (Myr)
*Neomys* rates, IM			
Introns + cytochrome *b*	0.40 (0.26 – 0.86)	0.56 (0.40 – 1.04)	1.22 (0.90 – 1.62)
Introns	0.43 (0.17 – 1.07)	0.61 (0.38 – 1.23)	1.50 (1.10 – 2.18)
Mammalian rates, IM			
Introns + cytochrome *b*	0.68 (0.44 – 1.47)	0.96 (0.69 – 1.77)	2.09 (1.54 – 2.76)
*Neomys* rates, I			
Introns + cytochrome *b*	0.27 (0.19 – 0.40)	0.46 (0.33 – 0.62)	1.14 (0.86 – 1.52)
*Neomys* rates, *BEAST			
Introns + cytochrome *b*	0.30 (0.19 – 0.43)	0.52 (0.35 – 0.71)	1.26 (0.87 – 1.70)
Gene tree approach, BEAST			
Introns	1.04 (0.64 – 1.48)	1.63 (1.12 – 2.22)	4.31 (3.11 – 5.57)

To check if the inclusion of cytochrome *b* greatly affected the IMa2 time estimations, we performed an additional analysis of the species tree parameters using only introns (Table 4). This analysis yielded slightly larger split times, particularly for the oldest split, but the overall values remained similar.

The use of mutation rates not specifically estimated for *Neomys* may have important consequences when calculating divergence times. To illustrate this effect, we used the mutation rates estimated for the entire mammalian tree in the Bayesian analysis of mammalian introns and recalculated split times with IMa2. As expected, using these lower rates resulted in older estimates of the split times (Table 4).

The importance of taking migration into account in the estimation of split times becomes evident when the results of the migration model were compared to the isolation model (without migration), using IMa2. As expected, all split times became smaller with respect to the isolation-with-migration model. Particularly, the split time of *N. a. anomalus* and *N. a. milleri* suffered proportionally the largest shift in the model without migration (Table 4). For comparison, we also used *BEAST (Table 4), which does not include migration in the model either. The results with *BEAST were similar to those obtained with IMa2 without migration. In addition, both IMa2 without migration and *BEAST yielded similar confidence intervals for all dates despite the fact that only the latter method used the variance of the mutation rates in the prior distributions (see Additional file 1).

Finally, we also evaluated the use of a direct gene tree approach (implemented in BEAST) for dating the split times of the *Neomys* lineages. To check this, we included the intron sequences of one specimen each of *N. a. anomalus, N. a. milleri* and *N. teres* with the other mammalian orthologues in a new BEAST analysis. As expected for nodes of nuclear gene trees younger than around 10 Myr [13], all split times were highly overestimated due to the effect of coalescence (Table 4).

## Discussion

### Effect of mutation rates and species tree models in split time estimates

The variability of the molecular clock and the effects of these variations in dating phylogenies have been known for a long time [10, 60]. In particular, the use of relaxed clock models in combination with multiple fossil calibrations has led to the reconstruction of highly reliable dated phylogenies [15, 16, 61]. Knowledge of higher-order phylogenies, like the phylogeny of mammals [62], has especially benefited from this strategy. However, there are additional issues that arise in the phylogenetic analyses of closely related species, particularly when applying coalescent-based species tree models, which have been less often covered [10]. A first problem when dating species trees of closely related species is that finding reliable internal fossil calibrations for the specific group of interest may not be possible. Here we tested a strategy in which lineage-specific mutation rates of the nuclear genes are estimated from a relaxed clock analysis of the same genes in a tree of mammals that included a set of reliable fossil calibrations. Following this approach, we estimated a mean substitution rate of 0.0060 substitutions/site/Myr for the introns in the *Neomys* lineage. This value is around twice as high as the mean rate of the introns averaged across all mammalian lineages. Consequently, if the mammalian rate was used, split times would be overestimated by a factor of two (Table 4). The extrapolation of the rate from a slower evolving group, such as humans, would lead to an even greater overestimation. In addition, it should be noted that intron rates have a large variation interval, with the most rapidly evolving intron being 5 times faster than the slowest one (Table 3). Therefore, if a different intron set is used, the variability of mutation rates should also be taken into account. As a consequence, it is most convenient to determine the rate for each locus and not simply to estimate averages of all loci. In conclusion, the uncritical extrapolation of mutation rates extracted from the literature for a different group and/or loci may lead to important errors in dating estimates of species trees.

Our approach to estimate mutation rates in mammals is not without problems. Most importantly, the dates of the calibrated nodes are quite old [15] in relation to the lineage of interest, *Neomys*, and therefore any change in the substitution rate before this lineage would remain undetected, even with the use of a relaxed clock model. In addition, the intron markers we used were originally selected for their high rates (together with other useful properties for phylogenetic reconstruction) [35]. Therefore, the same high rates that are most useful in the phylogenetic reconstruction of closely related species could be a potential problem for accurate substitution rate estimation from the tree of mammalian orders due to saturation. Without doubt, a greater effort in searching for a set of highly reliable fossils in additional branches of the tree of mammals like the Eulipotyphla, which contains *Neomys*, would be highly desirable for estimating more accurate rates in this order [15].

A different approach that can be used when internal calibrations are not available is the use of secondary calibrations obtained from a relaxed clock tree rather than extracting substitution rates from it. However, these estimated split times, if they are very recent, might be overestimated due to coalescence [13, 14]. In our example, all split times obtained from the BEAST gene tree of mammals were around three-fold overestimated (Table 4). If one of these split times was used as a secondary calibration for dating the species tree, the remaining nodes would be equally overestimated. It should be therefore preferable extracting substitution rates from the relaxed clock analysis.

Another aspect that should be taken into account for estimating accurate node ages is the possible presence of gene flow between lineages. Simulations have shown that gene flow may lead to highly underestimated split times if this parameter is not considered [63, 64]. The two *N. anomalus* lineages in which we were primarily interested revealed a small amount of gene flow between them. However, even with this small value, the use of a model with no gene flow would lead to a substantial underestimation (1.5×) of the split time of the two *N. anomalus* lineages. Therefore, if accurate split times are desired, models that take gene flow into account should be used, especially between lineages that are not completely isolated, which is the most likely scenario for shallow divergences.

### Loci and specimens used

We were interested in discerning the taxonomic status of two currently recognized subspecies of *N. anomalus: N. a. anomalus* and *N. a. milleri*. We used a large number of loci in order to apply sophisticated multilocus methods of species delimitation and split time estimation. This choice necessarily limited the number of individuals used in the multilocus analysis. However, the total number of alleles sampled from different loci is also an important aspect in these multilocus analyses. Simulation studies showed that the BPP method of Bayesian species delimitation is very robust with the use of 10 loci and 5 sequences per locus and per species (i.e., 50 sequences per species) [9]. In our case, we used 14 highly informative loci (including cytochrome *b*) and, in total, we obtained 86 and 102 sequences for *N. a. anomalus* and *N. a. milleri*, respectively, much more than the minimum theoretical optimum. Actually, the good convergence we achieved in BPP (as well as in IMa2) is likely due to the adequate number of loci and alleles per locus used. Of course, if the populations are highly structured, even these sequences may not encompass the total variability within each lineage. However, the specimens that we selected from both lineages cover a large part of their respective geographical distributions, including samples from the edges of their ranges. We also included several samples from close to the putative contact area between the two lineages, in the Iberian Peninsula, ruling out the possibility that the high divergence observed between *N. a. anomalus* ad *N. a. milleri* is due to an isolation-by-distance phenomenon. Also, these Iberian samples may have revealed specimens of problematic assignment if hybridization had been generalized. We can, therefore, be moderately confident that we have covered a significant proportion of the nuclear genetic variability within *N. anomalus*. Furthermore, the mitochondrial phylogeny, which includes a larger number of specimens, is very coherent with the multilocus analysis and revealed a strong genetic gap separating both lineages within the Iberian Peninsula (Figs. 1 and 2). However, denser sampling of nuclear data in the Iberian Peninsula should be carried out in order to better delimit the distribution of *N. a. anomalus* and *N. a. milleri* in the contact zone as well as to determine if there are areas of overlap or recent hybridization phenomena, which we have not detected so far. Additionally, according to the high genetic variability found in *N. a. milleri*, and that eastern and western specimens cluster into reciprocally monophyletic clades both in the mitochondrial and genome trees, it is possible that additional sampling in different parts of Europe may reveal the existence of other lineages within *N. a. milleri*.

### Group assignment

A first critical step in the multilocus study of a species complex is the assignment of individuals to different species or populations. Mitochondrial sequences are often used for this purpose in studies of cryptic species but the mitochondrial genome is among the most prone to introgression [65], so it is risky to use only these sequences for population assignment. As well as the mitochondrial tree (Fig. 2), we also reconstructed a tree based on the average genomic divergence (Fig. 3b). Trees based on different types of genome distance have been popularized in the study of archaic humans as a way to determine their genetic similarity to different hominins [66, 67]. These trees are affected by coalescence and cannot be used to extract demographic parameters, but they can reveal clusters of individuals with genomic similarity. In addition, we used Structurama [54], an objective method for delineating clusters based on allele frequencies. The four populations found by Structurama fit perfectly with the mitochondrial and genome trees and thus group assignment could be established with high confidence for all the samples.

### Species delimitation and taxonomy of *Neomys*

Coalescent-based analyses of multilocus data are starting to be considered as an objective way of defining species limits [4, 7]. These techniques are based explicitly or implicitly on the estimation of gene flow between lineages, which allows determining whether they have been evolving in isolation or not. Such lineages would correspond to separate species under a unified species concept [7, 68], in which separately evolving populations are treated as species, regardless of the properties acquired by lineages during the course of divergence (e.g., reproductive isolation, morphological diagnosability or niche differentiation). If intrinsic reproductive isolation develops during divergence, species would also be valid under a biological species concept. However, coalescence methods of species delimitation cannot prove reproductive isolation. In other words, these methods cannot support a biological species concept and therefore they are more directly related to a unified species concept [6, 7].

Using gene flow to determine lineage isolation is a clear advantage over genetic divergence, which has often been used for this purpose despite being only an indirect indication of isolation. It is well known that when the number of received effective migrants per generation (Nm) in a population is lower than 0.5, then the allele frequencies of the receiving population will vary from those of the source population due to random genetic drift [8]. By contrast, when Nm is higher than 0.5, genetic differentiation does not arise between the two populations. This reasoning, supported by empirical data, was used to propose an Nm threshold of 0.5 as a condition for the existence of species [5]. In the case of the *N. anomalus* subspecies, the migration parameters were much smaller than this threshold (0 and 0.072 for both migration directions, respectively), which would be compatible with the existence of two species. The BPP species delimitation test collapses two lineages when they do not fit a model of two isolated species. In fact, simulations with different degrees of gene flow showed an appropriate behavior of BPP with respect to the levels of migration rate known to cause population differentiation [9]. It is therefore not surprising that BPP supported the existence of two species within *N. anomalus.*

The different multilocus analysis methods used here support the fact that *N. a. anomalus* and *N. a. milleri* are independently evolving lineages and, therefore, according to these genetic results and following a unified species concept [7], they should be treated as distinct species, as originally described [23, 24]: *N. anomalus* (*sensu stricto*) and *N. milleri*. The distributions of the two species according to our current data are shown in Fig. 1: *N. anomalus* would be distributed across the Iberian Peninsula, with the exception of the northeastern corner, whereas *N. milleri* would occupy this part of the Iberian Peninsula and the rest of Europe and the Middle East. These distributions include the corresponding type localities of the original descriptions (Fig. 1). These two taxa were described in 1907 as morphological species and, therefore, the support for their species status comes, not only from the multilocus analysis performed here, but also from previously described morphological differences [23–27]. However, a thorough morphometric study should be carried out to better understand phenotypic differences between these two species. Nevertheless, it should not be discarded that character displacement or convergence due to competition in sympatry with the widely distributed *N. fodiens* [31, 32] may hinder the identification of unambiguous diagnostic morphological characters. The appropriate recognition of the independently evolving species within this genus will certainly help disentangle these ecological interactions.

### Using accurate split times to frame speciation

The split time of *N. anomalus* and *N. milleri* was estimated at 0.40 Myr ago and had a 95 % confidence interval of 0.26 – 0.86 Myr (Table 4 and Fig. 5) indicating that, most probably, it took place some time during the Middle Pleistocene (0.13 – 0.78 Myr) or, at most, at the end of the Early Pleistocene. During this part of the Pleistocene, glacial cycles had an average duration of around 100,000 years. Indeed, during the period that encompasses the 95 % confidence interval of the *N. anomalus* and *N. milleri* split, seven cold periods can be distinguished according to the marine isotope stages [69]. Therefore, we cannot identify at present with enough confidence in which specific glacial cycle of the Middle Pleistocene these species started to diverge. However, the confidence intervals associated to the split time allow rejecting that the speciation of *N. anomalus* and *N. milleri* occurred during the Pliocene, ending around 2.58 Myr, or during the Late Pleistocene, which started about 0.13 Myr ago and was dominated by a major glaciation in the Northern Hemisphere. The use of genome-wide data in the future may allow to narrow confidence intervals to the point that more specific periods within the Pleistocene can be associated with the divergence of sister species.

Based on the estimated split between *N. anomalus* and *N. milleri* during the Middle Pleistocene, a scenario of allopatric speciation linked to glacial cycles can be proposed for these two sister species. According to the extant species distributions, it is most likely that the ancestral population of *N. anomalus* became isolated in the Iberian Peninsula during one of the Middle Pleistocene glacial periods. As has been shown for several other small mammals [70], populations in this Mediterranean refugium would not have contributed to the recolonization of Europe during interglacial periods probably due to the limited dispersal imposed by the Pyrenean mountains as well as by the arid conditions at the south of these mountains [71], highly unsuitable for a semi-aquatic mammal*.* There is not enough evidence to know which refugia may have been occupied by *N. milleri* but, in contrast to the more restricted dispersal of *N. anomalus*, it would have arrived in the Iberian Peninsula through some corridors during the Holocene and possibly other interglacial periods. Actually, if speciation between *N. anomalus* and *N. milleri* was not associated to the last glaciation but started at an earlier glaciation, as our dating analysis shows, this would suggest that population expansions during successive interglacial periods may have occurred but did not result in complete admixture of the *Neomys* lineages. Only some interbreeding may have occurred during range expansions associated to warmer periods, explaining the small degree of gene flow observed in the multilocus analyses. However, it seems that this level of mixing was not enough to erode the specific identity of *N. anomalus* and *N. milleri*, as indicated by the results of the species delimitation test.

## Conclusions

Multilocus species trees combined with the estimation of accurate split times may help in advancing our knowledge of speciation. We showed here that the estimation of specific mutation rates for each locus and for the lineage of interest is a crucial aspect of a robust dating strategy since the extrapolation of mutation rates from other mammalian groups and loci may lead to very different split time estimates. In addition, the use of models that do not take coalescence and gene flow into account may also lead to biased results. We evaluated this strategy with several species and subspecies of the genus *Neomys* at the same time that we tried to resolve some controversial aspects of the taxonomy of this group. A Bayesian species delimitation method and estimation of gene flow supported that two subspecies deserve species status, as originally described: *N. anomalus* and *N. milleri*. In addition, the application of the dating methodology proposed here allowed the estimation of the split time between these two species in the Middle Pleistocene, which was compatible with a long period of isolation likely encompassing several glacial cycles. Therefore, obtaining accurate split times and associated confidence intervals in the species tree allows distinguishing between different Pleistocene ages and can provide important information for studies on recent speciation. Even if these times are not directly used in species delimitation, they allow testing explicit hypotheses about the geological and climatic context in which speciation occurred, thus helping to reconstruct more reliable scenarios on the initial stages of population divergence.

## Additional file

Additional file 1:
**Table S1.** *Neomys* specimens used, locations and number of genes sequenced for the species tree. **Table S2.** Cytochrome *b* sequences downloaded from GenBank. **Table S3.** Primers used for the amplification of three overlapping fragments of the mitochondrial cytochrome *b* gene. **Table S4.** Nuclear intron markers and primers used in this study. **Table S5.** GenBank accession numbers. **Table S6.** Calibration constraints (in Myr) used as priors in the BEAST analysis of mammalian introns. **Table S7.** Calibration constraints (in Myr) used as priors in the BEAST analysis of cytochrome *b* of soricids. **Figure S1.** Maximum-likelihood trees reconstructed from each individual intron of *Neomys*. **Figure S2.** Maximum-likelihood trees reconstructed from different concatenations of *Neomys* introns. **Figure S3.** Species tree obtained by *BEAST with branch lengths in relative units. **Figure S4.** Bayesian relaxed clock tree reconstructed with cytochrome *b* sequences of soricids. (PDF 1844 kb)
