# Supplementary material for: Multilocus species trees and species delimitation in a temporal context: application to the water shrews of the genus Neomys
Source: BMC Evol Biol. 2015 Sep 29;15:209. doi: 10.1186/s12862-015-0485-z (PMC4587729; doi:10.1186/s12862-015-0485-z)
Supplement: Additional file 1: — Table S1. Neomys specimens used, locations and number of genes sequenced for the species tree. Table S2. Cytochrome b sequences downloaded from GenBank. Table S3. Primers used for the amplification of three overlapping fragments of the mitochondrial cytochrome b gene. Table S4. Nuclear intron markers and primers used in this study. Table S5. GenBank accession numbers. Table S6. Calibration constraints (in Myr) used as priors in the BEAST analysis of mammalian introns. Table S7. Calibration constraints (in Myr) used as priors in the BEAST analysis of cytochrome b of soricids. Figure S1. Maximum-likelihood trees reconstructed from each individual intron of Neomys. Figure S2. Maximum-likelihood trees reconstructed from different concatenations of Neomys introns. Figure S3. Species tree obtained by *BEAST with branch lengths in relative units. Figure S4. Bayesian relaxed clock tree reconstructed with cytochrome b sequences of soricids. (PDF 1844 kb) [file 12862_2015_485_MOESM1_ESM.pdf]

Supplementary Methods, Tables and Figures

Multilocus species trees and species delimitation in a temporal context: application to the water shrews of the genus *Neomys*

Javier Igea, Pere Aymerich, Anna A. Bannikova, Joaquim Gosálbez and Jose Castresana

Supplementary Methods

Bayesian phylogenetic analyses of cytochrome *b*

Using BEAST version 1.8, the Markov chain was run for 50 million generations and 10% of the generations were discarded as burn-in. The program Tracer of the BEAST package was used to check that the effective sample sizes of all the parameters of interest were above 200 and convergence had been reached. TreeAnnotator of the same package was used to obtain the maximum clade credibility tree and the corresponding posterior probabilities of each clade.

Tree based on average genomic divergence

We calculated pairwise distances between all specimens using the formula 8.2 in Freedman et al. [1]. Basically, for each position, the average of the four possible matches between two individuals (one with nucleotides a and b and the other with nucleotides c and d) is computed as: $1 - (\delta_{ac} + \delta_{bd} + \delta_{ad} + \delta_{bc})/4$, where δ equals 1 if both nucleotides in the comparison are identical and 0 otherwise. Using a custom-made Perl script, this value was computed for all positions of all concatenated introns, summed, and divided by the total length to obtain the

distance between two individuals. A similar tree was obtained from distances calculated with formula 8.1 in Freedman et al. [1], where a more conservative estimate of the differences at each position is computed (not shown).

Structurama MCMC chain parameters

In each run, the Markov chain was run for 10 million generations, sampling every 100th cycle and with the initial 10,000 samples discarded as burn-in. Population assignment of each specimen was based on the mean partition or partition that minimizes the squared distance to all of the sampled partitions.

BEAST priors and MCMC chain parameters

Several mammalian fossil dates were used as hard bound minimum and soft bound maximum constraints in key nodes in order to calibrate the phylogenetic tree (Table S6). Specifically, we set lognormal prior distributions as follows: the offset was defined by the hard minimum, the mean in real space was adjusted so that the upper 95th percentile of the probability density distribution was coincident with the soft maximum, and the standard deviation parameter was set to 1 (Table S6). All calibrated nodes were older than 10 Myr, and therefore well above the time at which the difference between estimated gene tree and species tree divergences is minimal for nuclear genes [2]. A Yule speciation model was used as tree prior. 75 million generations were run and 10% of the generations were discarded as burn-in. The program Tracer of the BEAST package was used to check that the effective sample sizes of all the parameters of interest were above 200 and convergence had been reached.

For the soricid mitochondrial DNA analysis, the tree was calibrated using a set of fossil constraints available for soricids (Table S7), setting lognormal prior distributions as before.

All calibrated nodes were older than 3 Myr, and therefore well above the time at which the difference between estimated gene tree and species tree divergences is minimal for mitochondrial genes; this time is smaller than for nuclear genes due the reduced population size of mitochondrial genes [2]. To improve convergence, the priors of the substitution rate parameters of the GTR model and relative rate parameters of the codon positions were changed to uniform distribution between 0 and 100. 50 million generations were run, 10% of the generations were discarded as burn-in, and convergence was checked with Tracer.

***BEAST priors and MCMC chain parameters**

For each partition, HKY was selected as the substitution model. This model was used to match the model available in the program IMa2, which was used in a subsequent step to estimate additional parameters. However, it was checked that the use of more complex substitution models did not affect the results (not shown). The corresponding ploidy type of each marker (nuclear or mitochondrial) was set. In addition, a strict molecular clock was used for all partitions, a Yule process was set as species tree prior and the population size model was set as piecewise constant. All analyses were run for 50 million generations, 10% samples were discarded as burn-in and convergence was checked as before. The maximum clade credibility tree was constructed using median node heights.

IMa2 priors and MCMC chain parameters

The HKY model was used as substitution model. Maximum split time priors were set to 8, population size priors to 15 and migration rate priors to 2. Similar results were obtained when setting exponential migration rate priors with mean = 1 (not shown). When using cytochrome *b*, the heredity scalar for this locus was set as 0.25. Heating parameters were set as: hfg, hn15, ha0.96 and hb0.9. The final analyses consisted of a total of 50,000 sampled genealogies after

100,000 burn-in steps. As summary statistics of the posterior distributions, the bin with the highest value (after smoothing when this value was available in the IM output) and 95% confidence intervals were taken.

In IMa2, absolute mutation rates are not sampled in the MCMC chain. Rather, mutation rate scalars (the relative values of mutation rates) are estimated. The geometric mean of the externally estimated mutation rate of all loci is then used to scale demographic parameters, including divergence time. Therefore, unlike in *BEAST, it is not possible to introduce the variability of the rates that had been previously calculated in the mammalian multilocus analysis. However, it is possible to set ranges on mutation rates. The ratios of these limits are used as limits on the ratios of the mutation rate scalars. In order to test the effect of these limits, we used as mutation rate ranges the 95% confidence intervals of the mutation rates estimated in the previous mammalian multilocus analysis. In the introns-only analysis, the means and 95% confidence limits of the divergence times estimated by IMa2 were very similar than in the main analysis. When cytochrome *b* was included, the results with mutation rate ranges were more altered. However, these estimations were very similar again when the upper range of the cytochrome *b* was increased (10 times the estimated upper limit) to account for the possibility that the mutation rate previously calculated was saturated, similarly as we did in *BEAST (not shown).

BPP priors, MCMC chain parameters and additional tests

Mutation rates were set to be variable among loci and relative rates were generated from a Dirichlet distribution. When using cytochrome *b*, the heredity scalar for this locus was set as 0.25. As priors for θ (population size parameter) and τ (age of the root) we initially used values estimated from IMa2, after scaling them to reflect mutations per site. Two different

Gamma distributions were constructed for each of these parameters with $\alpha = 2$ (fairly diffused) and $\alpha = 20$ (more informative), respectively. The β of the Gamma distribution for θ and τ was obtained by dividing the α value by the corresponding mean of the parameter. Other divergence time parameters were assigned a Dirichlet prior. Additionally, we ran BPP with θ and τ priors that were respectively one order of magnitude lower and higher than the initial ones. Each prior set was analyzed using the two described reversible-jump Markov Chain Monte Carlo algorithms (rjMCMC) using default options. Each analysis consisted of 20,000 samples taken after 2000 burn-in steps.

References

1. Freedman AH, Gronau I, Schweizer RM, Ortega-Del Vecchyo D, Han E, Silva PM, Galaverni M, Fan Z, Marx P, Lorente-Galdos B, Beale H, Ramirez O, Hormozdiari F, Alkan C, Vilà C, Squire K, Geffen E, Kusak J, Boyko AR, Parker HG, Lee C, Tadigotla V, Siepel A, Bustamante CD, Harkins TT, Nelson SF, Ostrander EA, Marques-Bonet T, Wayne RK, Novembre J: **Genome sequencing highlights the dynamic early history of dogs**. *PLOS Genet* 2014, **10**:e1004016.

2. Sánchez-Gracia A, Castresana J: **Impact of deep coalescence on the reliability of species tree inference from different types of DNA markers in mammals.** *PLOS ONE* 2012, **7**:e30239.

Table S1. *Neomys* specimens used, locations and number of genes sequenced for the species tree.

Specimen Code	Species	Subspecies	Sample type	Locality (and map number)	Country	Lat.	Long.	Genes used in the species tree
IBE-C1529	*N. anomalus*	*anomalus*	Tissue (a)	Tielve (1)	Spain	43.3	-4.8	14
IBE-C1789	*N. anomalus*	*anomalus*	Tissue (b)	Navalguijo (2)	Spain	40.3	-5.5	14
IBE-C2895	*N. anomalus*	*anomalus*	Tissue (c)	Picos de Europa (3)	Spain	43.2	-4.9	14
IBE-C1435	*N. anomalus*	*anomalus*	Skull	Peñaflor de Hornija (4)	Spain	41.7	-5.0	1
IBE-C1683	*N. anomalus*	*anomalus*	Faeces	Trefacio (5)	Spain	42.2	-6.7	1
IBE-C1662	*N. anomalus*	*anomalus*	Faeces	Molinos de Razón (6)	Spain	42.0	-2.6	1
IBE-C1144	*N. anomalus*	*anomalus*	Faeces	Vega de Hórreo (7)	Spain	43.1	-6.6	1
IBE-C2664	*N. anomalus*	*anomalus*	Faeces	Bergantes (8)	Spain	40.7	-0.2	1
IBE-C1808	*N. anomalus*	*milleri*	Tissue (b)	La Pobla de Segur (9)	Spain	42.3	1.0	14
IBE-C3786	*N. anomalus*	*milleri*	Tissue (d)	Guardiola de Berguedà (10)	Spain	42.3	1.9	14
IBE-C4115 (S-168345)	*N. anomalus*	*milleri*	Tissue (e)	Vitebsk (11)	Belarus	55.2	30.2	9
IBE-C4116 (S-181445)	*N. anomalus*	*milleri*	Tissue (e)	Belgorod (12)	Russia	50.6	36.6	14
IBE-S1926	*N. anomalus*	*milleri*	Faeces	Osor (13)	Spain	41.9	2.5	1
IBE-C4120 (Nt49)	*N. teres*		Tissue (e)	North Caucasus (14)	Russia	43.9	40.1	14
IBE-C4122 (Nt77)	*N. teres*		Tissue (e)	North Caucasus (14)	Russia	43.9	40.1	14
IBE-C1914	*N. fodiens*		Tissue (f)	Coll	Spain	42.5	0.8	14
IBE-C101	*N. fodiens*		Tissue (b)	Queralbs	Spain	42.4	2.1	14
IBE-S1915	*N. fodiens*		Faeces	Zalduondo	Spain	42.9	-2.3	1

a, Capture permit CO/09/0004/2010, National Park Picos de Europa

b, Found dead in the field

c, Collection National Park Picos de Europa

d, Capture permit SF/209 (2012), Generalitat de Gatalunya

e, Previous work [1]

f, Capture permit SF/238 (2010), Generalitat de Gatalunya

[1] Bannikova, A. A., and D. A. Kramerov. 2005. Molecular phylogeny of Palearctic shrews inferred from RFLP and IS-PCR data. Advances in the biology of shrews II (eds. J. F. Merritt, S. Churchfield, R. Hutterer, and B. I. Sheftel). Special Publication of the International Society of Shrew Biologists 87–98.

Table S2. Cytochrome *b* sequences downloaded from GenBank.

GenBank Accession and reference	Species	Subspecies	Country (and map number)	Genes used in the species tree
DQ991052 [1]	*Neomys anomalus*	*milleri*	Italy (15)	1
DQ991049 [1]	*Neomys anomalus*	*milleri*	Italy (16)	1
DQ630409 [2]	*Neomys anomalus*	*milleri*	Macedonia (17)	
AF182182 [3]	*Neomys anomalus*	*milleri*	Turkey (18)	
AB175099 [4]	*Neomys anomalus*	*milleri*	Switzerland (19)	1
HQ621861 [6]	*Neomys teres*		Armenia (20)	1
HQ621860 [6]	*Neomys teres*		Armenia (20)	1
HQ621859 [6]	*Neomys teres*		Armenia (20)	1
HQ621858 [6]	*Neomys teres*		Armenia (20)	1
DQ991062 [1]	*Neomys fodiens*		Italy	1
AB175098 [4]	*Neomys fodiens*		Switzerland	1
AB175097 [4]	*Neomys fodiens*		Finland	1
AB175096 [4]	*Neomys fodiens*		Russia	1
AB175071 [5]	*Neomys fodiens*		China	1
GU981264 [7]	*Chimarrogale himalayica*		China	
GU981263 [7]	*Chimarrogale himalayica*		China	
AB108768 [8]	*Chimarrogale platycephala*		Japan	
AB108766 [8]	*Chimarrogale platycephala*		Japan	

References

1. Castiglia, R., Annesi, F., Aloise, G. & Amori, G. 2007 Mitochondrial DNA reveals different phylogeographic structures in the water shrews *Neomys anomalus* and *N. fodiens* (Insectivora: Soricidae) in Europe. *J. Zoolog. Syst. Evol. Res.* **45**, 255–262.

2. Dubey, S., Salamin, N., Ohdachi, S. D., Barrière, P. & Vogel, P. 2007 Molecular phylogenetics of shrews (Mammalia: Soricidae) reveal timing of transcontinental colonizations. *Mol. Phylogenet. Evol.* **44**, 126–137.

3. Kryštufek, B., Davison, A. & Griffiths, H. 2000 Evolutionary biogeography of water shrews (*Neomys* spp.) in the western Palaearctic Region. *Can. J. Zool.* **78**, 1616–1625.

4. Ohdachi, S. D., Hasegawa, M., Iwasa, M. A., Vogel, P., Oshida, T., Lin, L.-K. & Abe, H. 2006 Molecular phylogenetics of soricid shrews (Mammalia) based on mitochondrial cytochrome *b* gene sequences: with special reference to the Soricinae. *J. Zool.* **270**, 177–191.

5. Ohdachi, S. D., Vogel, P. & Ablimit, A. 2004 Mitochondrial cytochrome *b* sequence of *Neomys fodiens* from Xinjiang, China. *Unpublished*

6. Gajewska, M., Yavrouyan, E., Hayrapetian, W., Djavadian, R., Grigorian, M. & Turlejski, K. 2010 Low level of genetic polymorphism of shrews in Armenia and Nagorno-Karabakh. *Unpublished*

7. He, K., Li, Y.-J., Brandley, M. C., Lin, L.-K., Wang, Y.-X., Zhang, Y.-P. & Jiang, X.-L. 2010 A multi-locus phylogeny of Nectogalini shrews and influences of the paleoclimate on speciation and evolution. *Mol. Phylogenet. Evol.* **56**, 734–746.

8. Iwasa, M. A. & Abe, H. 2006 Colonization history of the Japanese water shrew *Chimarrogale platycephala*, in the Japanese Islands. *Acta Theriologica* **51**, 29–38.

Table S3. Primers used for the amplification of three overlapping fragments of the mitochondrial cytochrome *b* gene.

Primer	Sequence	Fragment
Neomys_tRNAGlu	ATCGTTGTTATTCAACTATAAGAAC	First
Neomys_cytb_403R	YCCYCARAATGATATTTGYCCTCA	First
Neomys_cytb_389F	GTTATAGCCACTGCCTTTATAG	Second
Neomys_cytb_746R	TAATTGTCCGGGTCTCCGAGTA	Second
Neomys_cytb_614F	TWTTCCTYCATGAAACAGGATC	Third
Neomys_tRNAThr	TTTTGGTTTACAAGACCAGTGTAT	Third

Table S4. Nuclear intron markers and primers used in this study. TD: touchdown PCR in which the annealing temperature was lowered from 65 ºC to 50 ºC at 1 ºC decrease per cycle.

Marker	Primer sequences	T (ºC)	Length
ALAD-10	AGAGTTYGCYATGYTGTGGCA / GGYGTGTAGTAGGTRATGATGA	TD	455
ASB6-2	TGYTGAAGATGGCYGAGCTG / TCCACCATGTCNGGCTGGTT	TD	319
CSF2-2	RAAACAGTARAWGTSRTCTCTG/ TNCAGACGNGTCTGCAGGCA	TD	673
CST6-1	RYTACAACATGGGCAGCAACA/ KGCMAGSGGGCARGTRGTGA	65	267
GALNT5-4	ATTYTTAGATTCTCAYGTGGAATG/ ACRTCYGGAGGAATKGTTCTC	60	727
GDAP1-1	ACDCATTCYTTCASYTCBCAAAAG/ CAAWGCCTTTTCAGCAATTACCA	TD	688
HIF1AN-5	TACGAGAGGTTYCCYAATTTCCA/ CTTATACCAGAAGTTCACAGTGAT	TD	389
JMJD-2	ACCABTGGCCVTGCATGMAGARGT/ TGATGAACTCRYTGACBGTCATGAG	TD	450
MCM3-2	GGAATTTATCAGAGCAAAGTTC/ RTAGAAYTCYTCRTACTGCTTG	TD	335
MYCBPAP-11	AAYAAYGGCACVGTGGYCATTT/ CAGCATYCRVAGAYTTRAAGAA	TD	341
PRPF31-3	GTCATYGTRGAYGCYAACAAC / BTTSACNGTGCGGATGTAATC	TD	481
SLA-2	AGGTGGCTGATGGCCTGTGCTGTG / TTCTTTCGATCAAAGGAGGTGTTGTC	TD	335
TRAIP-8	RGAGTAYGAGAAYCTDAAAGA / GCRCYCTGYAARTCCTTCTG	TD	685

Table S5. GenBank accession numbers

Accession numbers are given for the two alleles when available.

Specimen Code	Species	Subspecies	Cytochrome *b*	ALAD-10	ASB6-2	CSF2-2	CST6-1	GALNT5-4	GDAP1-1	HIF1AN-5	JMJD-2	MCM3-2	MYCBPAP-11	PRPF31-3	SLA-2	TRAIP-8
IBE-C1529	*Neomys anomalus*	*anomalus*	LK936659	LK936677, LK936678	LK936699, LK936700	LK936721, LK936722	LK936741, LK936742	LK936761, LK936762	LK936781, LK936782	LK936801, LK936802	LK936823, LK936824	LK936845, LK936846	LK936867, LK936868	LK936889, LK936890	LK936911, LK936912	LK936933, LK936934
IBE-C1789	*Neomys anomalus*	*anomalus*	LK936660	LK936679, LK936680	LK936701, LK936702	LK936723, LK936724	LK936743, LK936744	LK936763, LK936764	LK936783, LK936784	LK936803, LK936804	LK936825, LK936826	LK936847, LK936848	LK936869, LK936870	LK936891, LK936892	LK936913, LK936914	LK936935, LK936936
IBE-C2895	*Neomys anomalus*	*anomalus*	LK936661	LK936681, LK936682	LK936703, LK936704	LK936725, LK936726	LK936745, LK936746	LK936765, LK936766	LK936785, LK936786	LK936805, LK936806	LK936827, LK936828	LK936849, LK936850	LK936871, LK936872	LK936893, LK936894	LK936915, LK936916	LK936937, LK936938
IBE-C1144	*Neomys anomalus*	*anomalus*	LK936662													
IBE-C1435	*Neomys anomalus*	*anomalus*	LK936663													
IBE-C1662	*Neomys anomalus*	*anomalus*	LK936664													
IBE-C1683	*Neomys anomalus*	*anomalus*	LK936665													
IBE-C2664	*Neomys anomalus*	*anomalus*	LK936666													
IBE-C1808	*Neomys anomalus*	*milleri*	LK936667	LK936683, LK936684	LK936705, LK936706	LK936727, LK936728	LK936747, LK936748	LK936767, LK936768	LK936787, LK936788	LK936807, LK936808	LK936829, LK936830	LK936851, LK936852	LK936873, LK936874	LK936895, LK936896	LK936917, LK936918	LK936939, LK936940
IBE-C3786	*Neomys anomalus*	*milleri*	LK936668	LK936685, LK936686	LK936707, LK936708	LK936729, LK936730	LK936749, LK936750	LK936769, LK936770	LK936789, LK936790	LK936809, LK936810	LK936831, LK936832	LK936853, LK936854	LK936875, LK936876	LK936897, LK936898	LK936919, LK936920	LK936941, LK936942
IBE-C4115	*Neomys anomalus*	*milleri*	LK936669	LK936687, LK936688	LK936709, LK936710					LK936811, LK936812	LK936833, LK936834	LK936855, LK936856	LK936877, LK936878	LK936899, LK936900	LK936921, LK936922	
IBE-C4116	*Neomys anomalus*	*milleri*	LK936670	LK936689, LK936690	LK936711, LK936712	LK936731, LK936732	LK936751, LK936752	LK936771, LK936772	LK936791, LK936792	LK936813, LK936814	LK936835, LK936836	LK936857, LK936858	LK936879, LK936880	LK936901, LK936902	LK936923, LK936924	LK936943, LK936944
IBE-S1926	*Neomys anomalus*	*milleri*	LK936671													
IBE-C101	*Neomys fodiens*		LK936672	LK936691, LK936692	LK936713, LK936714	LK936733, LK936734	LK936753, LK936754	LK936773, LK936774	LK936793, LK936794	LK936815, LK936816	LK936837, LK936838	LK936859, LK936860	LK936881, LK936882	LK936903, LK936904	LK936925, LK936926	LK936945, LK936946
IBE-C1914	*Neomys fodiens*		LK936673	LK936693, LK936694	LK936715, LK936716	LK936735, LK936736	LK936755, LK936756	LK936775, LK936776	LK936795, LK936796	LK936817, LK936818	LK936839, LK936840	LK936861, LK936862	LK936883, LK936884	LK936905, LK936906	LK936927, LK936928	LK936947, LK936948
IBE-S1915	*Neomys fodiens*		LK936674													
IBE-C4120	*Neomys teres*		LK936675	LK936695, LK936696	LK936717, LK936718	LK936737, LK936738	LK936757, LK936758	LK936777, LK936778	LK936797, LK936798	LK936819, LK936820	LK936841, LK936842	LK936863, LK936864	LK936885, LK936886	LK936907, LK936908	LK936929, LK936930	LK936949, LK936950
IBE-C4122	*Neomys teres*		LK936676	LK936697, LK936698	LK936719, LK936720	LK936739, LK936740	LK936759, LK936760	LK936779, LK936780	LK936799, LK936800	LK936821, LK936822	LK936843, LK936844	LK936865, LK936866	LK936887, LK936888	LK936909, LK936910	LK936931, LK936932	LK936951, LK936952
IBE-C103	*Crocidura russula*				LK936954					LK936959	LK936961	LK936963	LK936965	LK936967		
IBE-S1189	*Crocidura russula*							LK936957								
IBE-C1918	*Sorex coronatus*					LK936955		LK936958						LK936968		LK936969
IBE-C1920	*Sorex coronatus*			LK936953			LK936956			LK936960	LK936962	LK936964	LK936966			

Table S6. Calibration constraints (in Myr) used as priors in the BEAST analysis of mammalian introns. Node numbers correspond to numbers in figure 4.

Clade (Node number)	Minimum hard bound	Maximum soft bound	Lognormal parameters	
			Mean	Offset
Boreoeutheria (1)	61.50	131.50	22.28	61.50
Laurasiatheria (2)	62.50	131.50	21.95	62.50
Eulipotyphla (3)	61.50	131.50	22.28	61.50
Ferungulata (4)	62.50	131.50	21.95	62.50
Zooamata (5)	62.50	131.50	21.95	62.50
Cetartiodactyla (6)	52.40	65.80	4.27	52.40
Carnivora (7)	39.68	65.80	8.28	39.68
Catarrhini (8)	23.5	34.00	3.35	23.50

Table S7. Calibration constraints (in Myr) used as priors in the BEAST analysis of cytochrome *b* of soricids. Node numbers correspond to numbers in figure S1.

Clade (Node number)	Minimum hard bound	Maximum soft bound	Lognormal parameters	
			Mean	Offset
Soricinae–Crocidurinae (1)	20	25	1.59	20
Blarinini (2)	15	20	1.59	15
Otisorex (3)	3.5	5	0.48	3.5

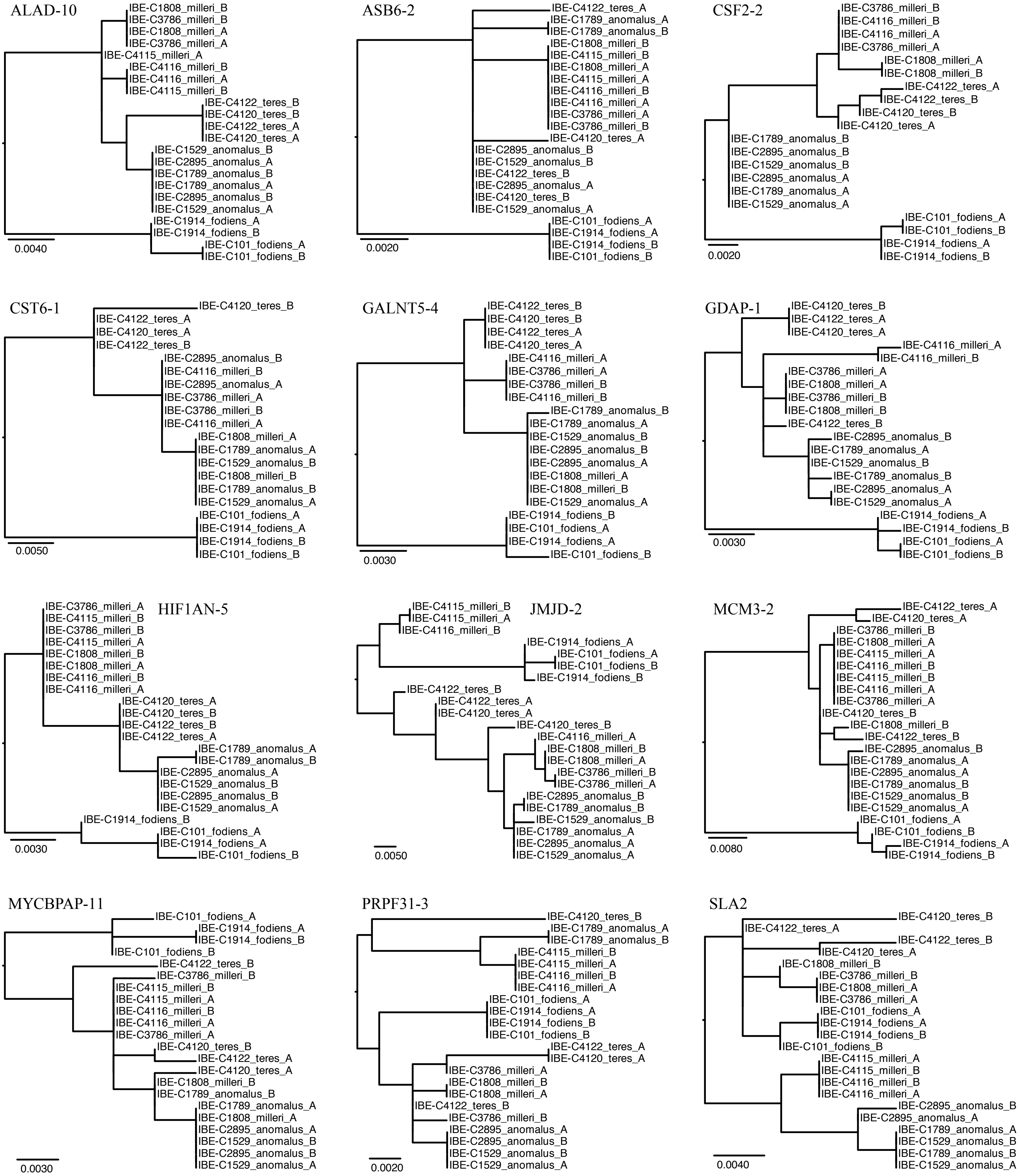

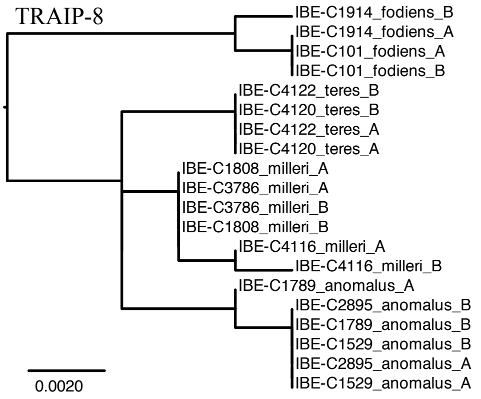

Figure S1. Maximum-likelihood trees reconstructed from each individual intron of *Neomys*. The two alleles of each specimen are indicated with the letters A and B. The trees were rooted at the midpoint. The scale is in substitutions/site.

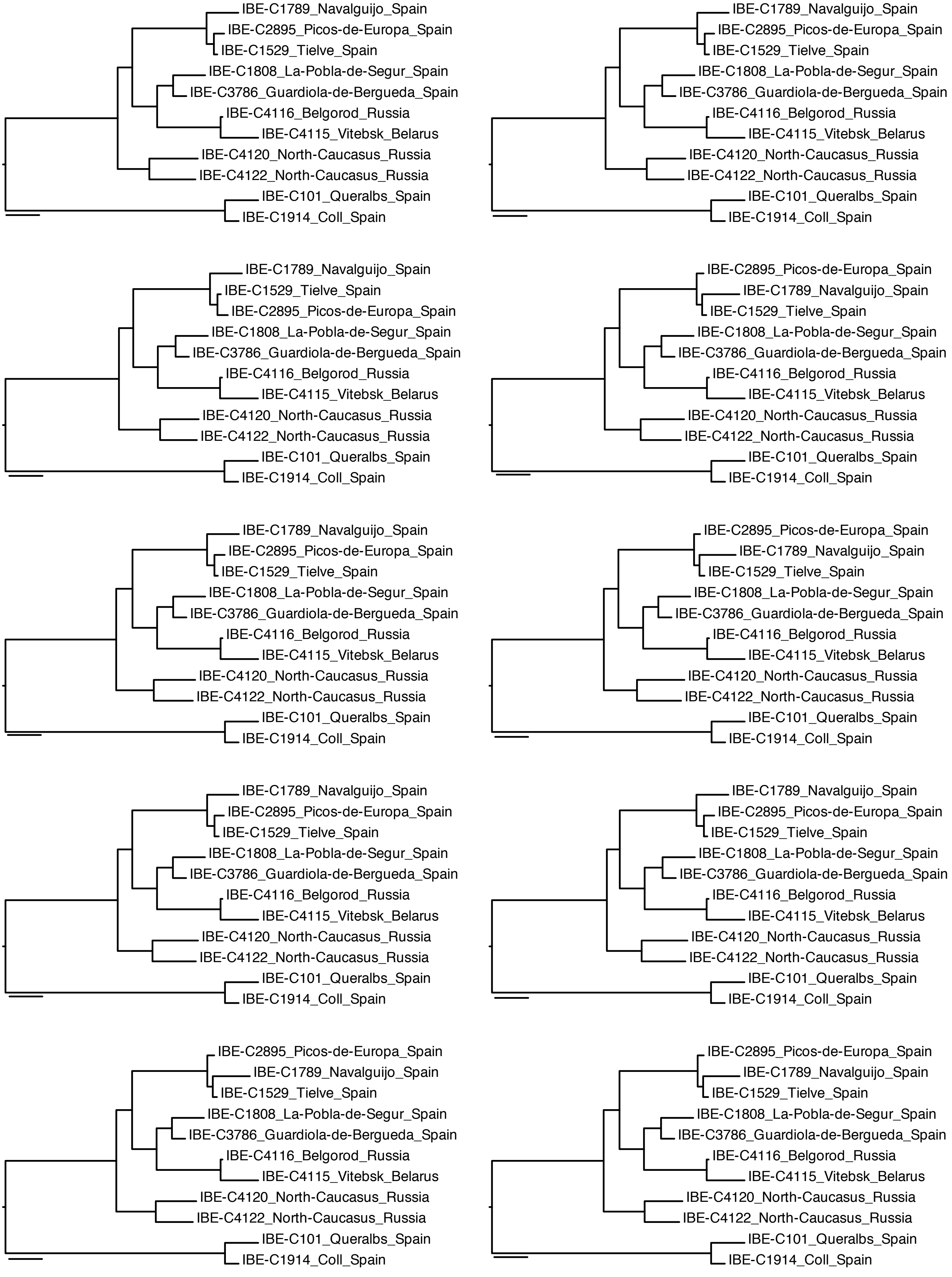

Figure S2. Maximum-likelihood trees reconstructed from different concatenations of *Neomys* introns. In each concatenation, the order of each allele pair was randomly changed. Names include specimen code and locality data. The trees were rooted at the midpoint. The scale bar represents 0.002 substitutions/site in all trees.

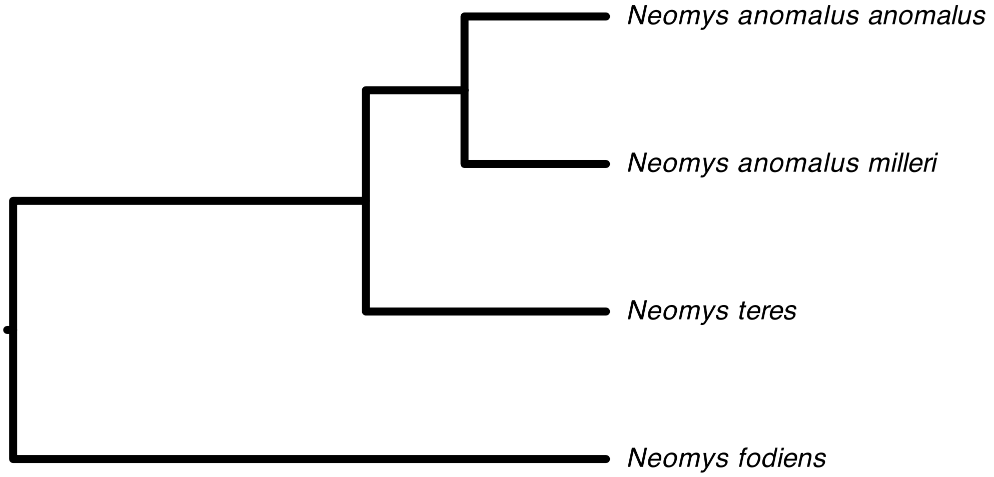

Figure S3. Species tree obtained by *BEAST with branch lengths in relative units.

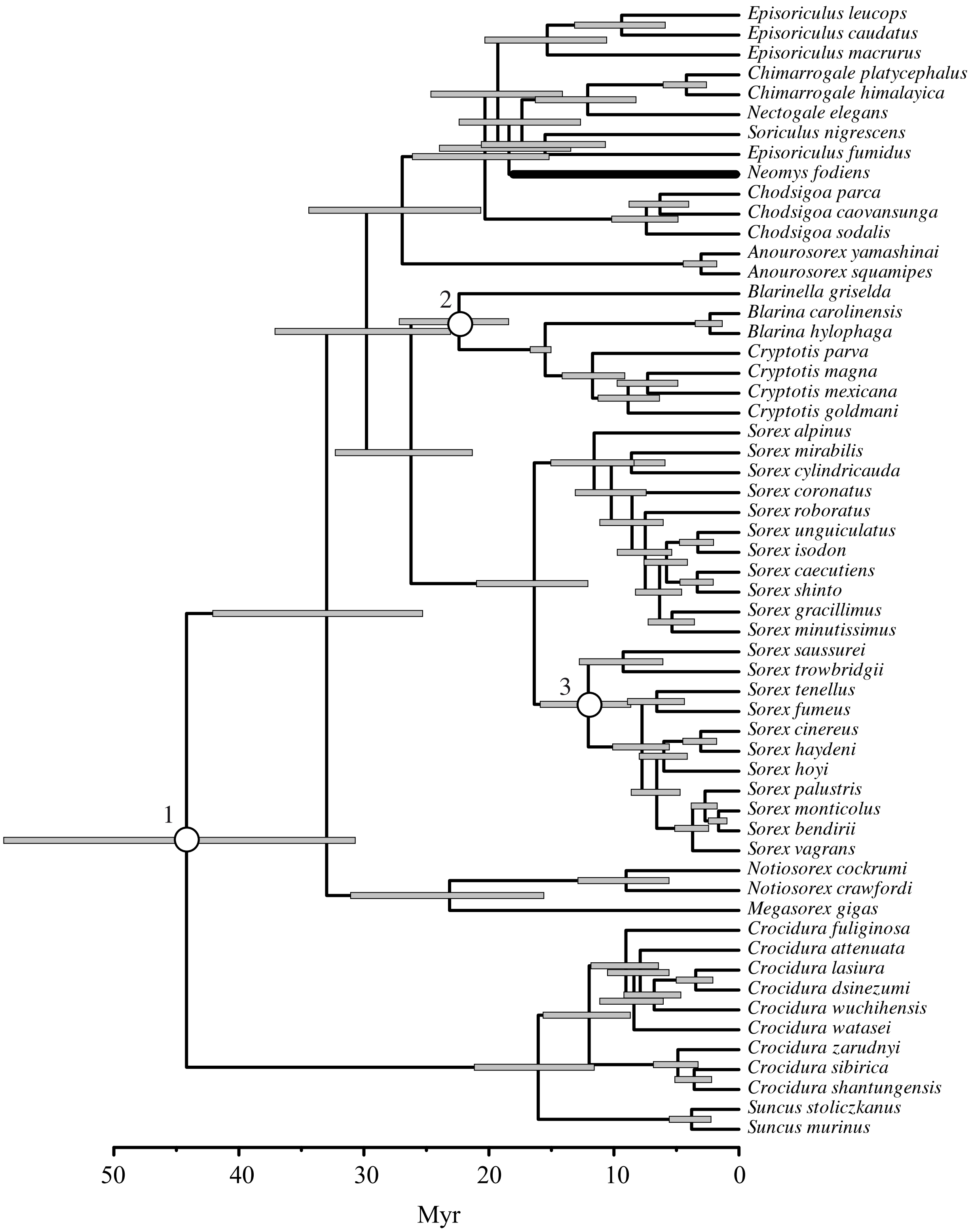

Figure S4. Bayesian relaxed clock tree reconstructed with cytochrome *b* sequences of soricids. Calibration nodes are shown with a white circle and the corresponding constraints are given in Table S7. The *Neomys fodiens* branch from which the mutations rate was estimated is shown with a thicker line.
